# Neutrophil–lymphocyte ratio (NLR), platelet–lymphocyte ratio (PLR) and lymphocyte–monocyte ratio (LMR) in predicting systemic inflammatory response syndrome (SIRS) and sepsis after percutaneous nephrolithotomy (PNL)

**DOI:** 10.1007/s00240-022-01319-0

**Published:** 2022-03-04

**Authors:** Akshay Kriplani, Shruti Pandit, Arun Chawla, Jean J. M. C. H. de la Rosette, Pilar Laguna, Suraj Jayadeva Reddy, Bhaskar K. Somani

**Affiliations:** 1 Department of Urology and Renal Transplant, Kasturba Medical College, Manipal Academy of Higher Education, Manipal, Karnataka India; 2 Istanbul Medipol Mega University Hospital, Istanbul, Turkey; 3 Department of Urology, University Hospital Southampton NHS Trust, Southampton, UK

**Keywords:** SIRS, PCNL, Sepsis, Renal stones, Endourology

## Abstract

The objective of this prospective observational study was to assess the clinical significance of neutrophil–lymphocyte ratio (NLR), platelet–lymphocyte ratio (PLR) and lymphocyte–monocyte ratio (LMR) as potential biomarkers to identify post-PNL SIRS or sepsis. Demographic data and laboratory data including hemoglobin (Hb), total leucocyte count (TLC), serum creatinine, urine microscopy and culture were collected. The NLR, LMR and PLR were calculated by the mathematical division of their absolute values derived from routine complete blood counts from peripheral blood samples. Stone factors were assessed by non-contrast computerized tomography of kidneys, ureter and bladder (NCCT KUB) and included stone burden (Volume = L × W × D × π × 0.167), location and Hounsfield value and laterality. Intraoperative factors assessed were puncture site, tract size, tract number, operative time, the need for blood transfusion and stone clearance. Of 517 patients evaluated, 56 (10.8%) developed SIRS and 8 (1.5%) developed sepsis. Patients developing SIRS had significantly higher TLC (10.4 ± 3.5 vs 8.6 ± 2.6, OR 1.19, 95% CI 1.09–1.3, *p* = 0.000002), higher NLR (3.6 ± 2.4 vs 2.5 ± 1.04, OR 1.3, 95% CI = 1.09–1.5, *p* = 0.0000001), higher PLR (129.3 ± 53.8 vs 115.4 ± 68.9, OR 1.005, 95% CI 1.001–1.008, *p* = 0.005) and lower LMR (2.5 ± 1.7 vs 3.2 ± 1.8, OR 1.18, 95% CI 1.04–1.34, *p* = 0.006). Staghorn stones (12.8 vs 3.24%, OR 4.361, 95% CI 1.605–11.846, *p* = 0.008) and long operative times (59.6 ± 14.01 vs 55.2 ± 16.02, OR 1.01, 95% CI 1.00–1.03, *p* = 0.05) had significant association with postoperative SIRS. In conclusion, NLR, PLR and LMR can be useful independent, easily accessible and cost-effective predictors for early identification of post-PNL SIRS/sepsis.

## Introduction

Percutaneous nephrolithotomy (PNL) is the surgical option of choice for upper urinary tract calculi of size > 2 cm and selected calculi < 2 cm [1]. Clinical spectrum of infective complications post-PNL range from transient fever to sever sepsis, with urosepsis reported in 0.9 to 4.7% of PCNL procedures [2–4]. Sepsis is also considered one of the most common causes of perioperative mortality after PNL [2, 5]. Female gender, diabetes, pyuria, large stone, staghorn stone, infected stone, positive pelvic urine culture and use of nephrostomy tube [6–11] are important predictors of postoperative SIRS. This study is aimed to assess the clinical significance of neutrophil–lymphocyte ratio (NLR), platelet–lymphocyte ratio (PLR) and lymphocyte–monocyte ratio (LMR) as potential biomarkers to identify post-PNL SIRS or sepsis.

## Patients and methods

After institutional ethics committee approval and registration with Clinical Trial registry of India (REF/2018/09/021711), we did a prospective observational study of consecutive patients undergoing PNL at the urology department of a tertiary referral center from Karnataka, India, between November 2018 and October 2019. Standard protocols were followed for evaluation, treatment and follow-up. Demographic data collected were age, body mass index (BMI), comorbidities including hypertension, diabetes mellitus and history of previous PNL. Laboratory data included hemoglobin (Hb), total leucocyte count (TLC), serum creatinine, urine microscopy and culture. The NLR, LMR and PLR were calculated by the mathematical division of their absolute values derived from routine complete blood counts from peripheral blood samples on the day prior to surgery. Stone factors were assessed by non-contrast computerized tomography of kidneys, ureter and bladder (NCCT KUB) and included stone burden (Volume = L × W × D × π × 0.167), location and Hounsfield value and laterality. Intraoperative factors assessed were puncture site, tract size, tract number, operative time, the need for blood transfusion, stone clearance, usage of ureteral stent or nephrostomy tube and any ancillary procedures. The operative procedure followed a standardized prone PNL protocol, under general anesthesia and IV third-generation cephalosporin at induction. A sterile preoperative urine culture was ensured in all patients. Postoperative blood parameters included Hb, TLC, and serum creatinine as per the clinical condition. Analgesia was provided using parenteral tramadol. Postoperative complications were documented using the modified Clavien–Dindo grading system [12]. Patients with up to Grade 1 complications were discharged on postoperative day 2. Postoperative fever was defined as temperature > 38 °C. Systemic inflammatory response syndrome (SIRS) was defined as the presence of two of more of the following parameters: body temperature > 38 °C or < 36 °C, heart rate > 90/min, respiratory rate > 20 breaths/min and white blood cell count > 12 × 10^9^ or < 4 × 10^9^ cells/L. Sepsis was defined as both SIRS and a positive postoperative blood or urine culture [13].

Statistical analysis was done on SPSS, version 16.0. Categorical variables were expressed in frequencies with percentages and compared using Chi-square or Fisher’s exact test, continuous variables as mean and standard deviation and compared using Student’s *t* test for those with normal distribution and median with interquartile range with comparison using Mann–Whitney test for those with skewed distribution and a *p* value ≤ 0.05 considered significant. Univariate analysis was done to check the relation between the dependent variable (occurrence of SIRS/sepsis) and each of the independent variables. Multivariate analysis was then performed using logistic regression to establish the predictive factors for the development of AKI. A receiver operating characteristic (ROC) curve was constructed, and area under curve was used to derive a cutoff value for the variable.

## Results

Of 517 patients evaluated, mean age was 48.1 ± 13.9 years, 56 (10.8%) developed post-PNL SIRS and 8 (1.5%) developed sepsis. The details of patient demography and stone characteristics with the univariate analysis for predictive factors for development of postoperative SIRS and sepsis are mentioned in Tables 1 and 2.

**Table 1:** Patient characteristics, preoperative laboratory values and stone characteristics

Variables	All patients (*n* = 517)	SIRS ( ± ) (*n* = 461)	SIRS (+) (*n* = 56)	*p* value	Sepsis (−) (*n* = 509)	Sepsis (+) (*n* = 8)	*p* value
Patient characteristics							
Age (years) (mean ± SD)	48.13 ± 13.92	47.5 ± 13.8	46.5 ± 14.5	0.6	47.4 ± 13.9	45 ± 13.3	0.6
Gender							
Female	127 (24.6%)	112 (88.2%)	15 (11.8%)	0.68	126 (99.2%)	1 (0.8%)	0.42
Male	390 (75.4%)	349 (89.5%)	41 (10.5%)		383 (98.2%)	7 (1.8%)	
BMI (kg/m^2^)	25.23 ± 2.94	25.2 ± 2.93	25.3 ± 2.9	0.7	25.1 ± 2.9	27.3 ± 1.9	0.03*
Hypertension	145 (27.9%)	120 (26%)	14 (25%)	1.0	132 (26%)	2 (25%)	1.0
Diabetes mellitus	96 (18.5%)	82 (17.8%)	12 (21.4%)	0.4	90 (17.7%)	4 (50%)	0.04
History of previous ipsilateral PCNL (%)	23 (4.4%)	19 (4.1%)	4 (7.1%)	0.3	23 (4.5%)	0	1.0
Preoperative laboratory values							
Hemoglobin (gm/dl)	13.29 ± 1.91	13.4 ± 1.8	12.8 ± 2.3	0.04*	13.3 ± 1.9	12.9 ± 2.5	0.6
TLC (/mm3)	8.73 ± 3.84	8.6 ± 2.6	10.4 ± 3.5	0.000002*	8.8 ± 2.8	9.5 ± 2.3	0.4
Creatinine (mg/dl)	1.42 ± 4.30	1.4 ± 4.5	1.2 ± 0.7	0.75	1.4 ± 4.3	1.03 ± 0.3	0.8
NLR	2.6 ± 2.1	2.5 ± 1.04	3.6 ± 2.4	0.0000001*	2.6 ± 2.1	3.9 ± 5.7	0.2
PLR	118.3 ± 69.5	115.4 ± 68.9	129.3 ± 53.8	0.005*	118.2 ± 69.7	143.2 ± 70.03	0.6
LMR	2.6 ± 1.7	3.2 ± 1.8	2.6 ± 1.7	0.006*	4.07 ± 2.8	2.5 ± 1.7	0.02*
Stone characteristics							
Stone volume (mm^3^) [median (Q1-Q3)]	880.95 (524.38–1801.25)	825 (503–1573)	890 (529–3089)	0.3	839 (515–1641)	849 (616–1090)	0.9
Hounsfield unit (HU)	970.59 ± 278.55	986 ± 253.1	1017 ± 294.4	0.39	753 ± 407	993 ± 253	0.009*
Staghorn (%)	4.06	3.24	12.8	0.008*	4.12	0	1.0
Stone location (%)							
Simple	87.8	88.1	85.7	0.6	87.6	100	0.6
Complex	12.2	11.9	14.3		12.4	0	
Intraoperative characteristics							
Puncture site (%)							
Supracostal (*n* = 75)	14.7	13.9	19.6	0.2	14.5	12.5	1.0
Infracostal (*n* = 442)	85.3	86.1	80.4		85.5	87.5	
Tract size [median (Q1-Q3)]	28 (26–32)	28 (26–32)	28 (26–32)	0.2	28 (26–32)	28 (15–32)	0.8
Bilateral puncture (%)	4.6	4.3	7.1	0.3	4.6	0	1.0
Tract number (%)							
Single puncture	97.6	97.4	100	0.3	97.6	100	1.0
> 1 puncture	2.35	2.6	0		2.4	0	
Blood transfusion (%)	2.9	2.6	7.1	0.08	2.9	12.5	0.2
Operative time (minutes)	55.99 ± 16.71	55.2 ± 16.02	59.6 ± 14.01	0.05*	55.7 ± 15.8	55 ± 17.3	0.8
LOH (days)	2.34 ± 1.78	2.22 ± 1.75	3.30 ± 1.71	0.00003*	2.34 ± 1.78	3.34 ± 1.69	0.001*

*Values are statistically significant

**Table 2:** Univariate and multivariate logistic regression analyses for predictors of post-PCNL SIRS

Variable	Univariate analysis		Multivariate analysis	
	Unadjusted OR	*p* value	Adjusted OR	*p* value
Patient characteristics				
Age	0.9 (0.97–1.01)	0.61	1.007 (0.96–1.04)	0.7
Gender				
Female	0.87 (0.46–1.6)	0.68	1.48 (0.39–5.6)	0.57
Male	1.0		1.0	
BMI	1.01 (0.92–1.11)	0.75	1.10 (0.92–1.31)	0.28
Hypertension				
Yes	0.94 (0.5–1.7)	0.86	1.03 (0.27–3.9)	0.95
No	1.0			
Diabetes mellitus				
Yes	1.2 (0.63–2.49)	0.5	0.29 (0.65–1.3)	0.1
No	1.0			
History of previous PCNL	1.7 (0.58–5.46)	0.3	1.0 (0.36–1.56)	0.01*
Preoperative laboratory values				
Hemoglobin	0.86 (0.7–0.9)	0.04*	0.74 (0.53–1.03)	0.07
TLC	1.19 (1.09–1.3)	0.00006*	1.22 (1.04–1.42)	0.01*
Creatinine	0.98 (0.85–1.12)	0.77	0.72 (0.17–2.96)	0.65
Preoperative pyuria	1.0	0.151	0.99 (0.99–1.002)	0.65
NLR	1.3 (1.09–1.5)	0.003*	1.65 (1.22–2.24)	0.001*
PLR	1.005 (1.001–1.008)	0.006*	1.008 (1.002–1.014)	0.008*
LMR	1.18 (1.04–1.34)	0.008*	1.27 (1.03–1.56)	0.025*
Stone characteristics				
Stone volume	1.0	0.8	1.0	0.84
Hounsfield unit (HU)	1.0 (0.99–1.002)	0.3	1.0	0.07
Staghorn	4.361 (1.605–11.846)	0.004*	0.594 (0.032–10.944)	0.726
Stone location (*n*)				
Simple	0.478 (0.394–1.548)	0.61	1.582 (0.205–12.207)	0.660
Complex				
Puncture site				
Supracostal (*n* = 75)	0.6 (0.32–1.34)	0.25	0.52 (1.38–1.95)	0.33
Infracostal				
Tract size	0.97 (0.92–1.02)	0.25	0.88 (0.69–1.12)	0.31
Bilateral puncture	1.6 (0.5–5.1)	0.3		
Simultaneous Ipsilateral URS(*n* = 442)	1.27 (0.28–5.8)	0.7	1.62 (0.10–24.8)	0.72
PNL type				
Standard	0.697 (0.31–1.56)	0.38	0.720 (0.024–21.4)	0.85
Mini PNL				
Tubeless				
Operative time	1.01 (1.00–1.03)	0.05*	1.02 (0.98–1.06)	0.25
LOH	1.31 (1.15–1.49)	0.00004*	1.46 (1.16–1.83)	0.001*

*Values are statistically significant

Patients developing SIRS had significantly lower preoperative hemoglobin (12.8 ± 2.3 vs 13.4 ± 1.8, OR 0.86, 95% CI 0.7–0.9, *p* = 0.04), higher TLC (10.4 ± 3.5 vs 8.6 ± 2.6, OR 1.19, 95% CI 1.09–1.3, *p* = 0.000002), higher NLR (3.6 ± 2.4 vs 2.5 ± 1.04, OR 1.3, 95% CI 1.09–1.5, *p* = 0.0000001), higher PLR (129.3 ± 53.8 vs 115.4 ± 68.9, OR 1.005, 95% CI 1.001–1.008, *p* = 0.005) and lower LMR (2.5 ± 1.7 vs 3.2 ± 1.8, OR 1.18, 95% CI 1.04–1.34, *p* = 0.006). Staghorn stones (12.8 vs 3.24%, OR 4.361, 95% CI 1.605–11.846, *p* = 0.008) and long operative times (59.6 ± 14.0 vs 55.2 ± 16.0, OR 1.01, 95% CI 1.00–1.03, *p* = 0.05) had significant association with postoperative SIRS. Length of hospital stay (days) was significantly more in the SIRS cohort (3.3 ± 1.7 vs 2.2 ± 1.7, OR 1.31, 95% CI 1.15–1.49, *p* = 0.00003). On multivariable logistic regression analysis, the independent risk factors for SIRS were history of previous ipsilateral PNL (OR 1.0, 95% CI = 0.36–1.56, *p* = 0.01), raised preoperative TLC (OR 1.22, 95% CI 1.04–1.42, *p* = 0.01), raised NLR (OR = 1.6, 95% CI = 1.22–2.24, p = 0.001), raised PLR (OR = 1.008, 95% CI = 1.002–1.014, *p* = 0.008) and low LMR (OR 1.3, 95% CI 1.03–1.56,* p* = 0.02).

Predictive values for sepsis post-PNL were high BMI (27.3 ± 1.9 vs 25.1 ± 2.9, *p* = 0.03), diabetes mellitus (50 vs 17.7%, *p* = 0.04), low LMR (2.6 ± 1.7 vs 4.1 ± 2.8, *p* = 0.02) and high stone density (993 ± 253 vs 753 ± 407, *p* = 0.009). All patients with sepsis were managed with intravenous antibiotics as per culture sensitivity and no patients required intensive care support in our cohort. Sepsis significantly increased the length of hospital stay (days) (3.3 ± 1.7 vs 2.3 ± 1.8, OR 1.31, 95% CI 1.15–1.49, *p* = 0.001).

**Fig. 1:**
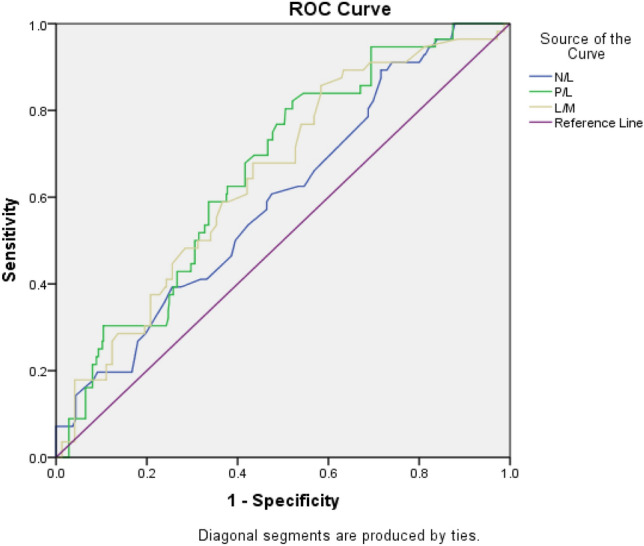
Receiver operating characteristic (ROC) curve analysis results of NLR, PLR and LMR in predicting postoperative SIRS

**Fig. 2:**
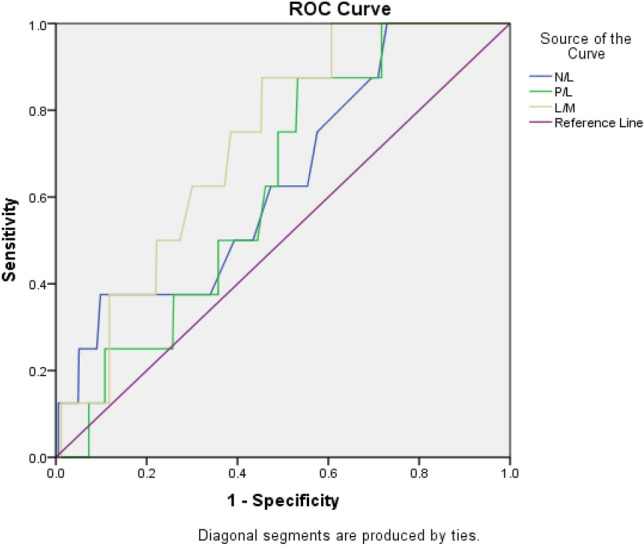
Receiver operating characteristic (ROC) curve analysis results of NLR, PLR and LMR in predicting postoperative sepsis

On ROC analysis, the cutoff for preoperative NLR to predict postoperative SIRS was 2.03 with 82% sensitivity and 31% specificity with the area under the curve of 0.596 (*p* = 0.018) (Fig. 1). Cutoff for postoperative sepsis was 2.45 with 87% sensitivity and 31% specificity with area under the curve of 0.639 (*p* = 0.17) (Fig. 1). The threshold for PLR for postoperative SIRS was 110.62 with 80.2% sensitivity and 50.5% specificity with area under curve of 0.663 (*p* = 0.00006). Threshold for postoperative sepsis was 120.25 with 87.5% sensitivity and 53.2% specificity with area under the curve of 0.627 (*p* = 0.21). The cutoff value for LMR for postoperative SIRS was 3.23 with 83.9% sensitivity and 42% specificity with area under the curve of 0.649 (*p* = 0.0002). Cutoff value for postoperative sepsis was 2.88 with 87.5% sensitivity and 55% specificity with area under the curve of 0.726 (*p* = 0.02) (Fig. 2).

In this cohort, 21/517 (4.1%) patients had staghorn calculi. Among them 7 patients (33.3%) developed SIRS and 14 patients (66.7%) did not develop SIRS. Out of the 496 (95.6%) non-staghorn patients, 49 (9.8%) developed SIRS and 447 (90.1%) did not develop SIRS (OR 4.56, 95% CI 1.75–11.84, *p* = 0.0018). In the staghorn cohort, sensitivity and specificity of high NLR (> 2.0), high PLR (> 110.6) and low LMR (< 3.2) to predict SIRS were 85.7% and 78.6%, 83.3% and 71.4% and 71.4% and 71.4%, respectively, whereas sensitivity and specificity of low hemoglobin (Hb < 120gm/L) were 57.1% and 71.4%.

## Discussion

Infection-related complications post-PNL range from transient fever to SIRS and sepsis. Incidence of fever post-PNL ranges from 10 to 25% [14, 15]. Incidence of SIRS in our cohort (10.83%) was much less than the reported incidence of 16.7—27.4% [2, 7, 10, 16–18]. This could be attributed to ensuring a preoperative sterile urine culture in all patients. In the largest prospective CROES study [19] evaluating the post-PNL complications, 10.5% patients developed fever, UTI in 0.6% and 0.2% progressing to sepsis.

Postoperative sepsis in the absence of bacteremia or bacteriuria could be hypothesized to persisting bacterial endotoxins in infected stones [11, 20, 21]. ‘Nanobacteria’ have also been theorized in stone formation and post-PNL infective complications [11, 22, 23]. Lack of a uniform consensus on the preoperative risk factors makes it imperative to identify a biomarker that is inexpensive and readily available. While TLC is a less sensitive marker, NLR, PLR and LMR can be useful in identifying systemic inflammation. These have been utilized in predicting prognosis in malignancies [24–27], inflammatory disorders [28], atherosclerosis [29], cardiovascular disorders [30] or metabolic syndrome [31, 32]. The role of these mediators in stone crystallization has established the immune response, oxidative stress and inflammatory cell response theory of stone formation, especially in patients with metabolic syndrome [33–35].

In our prospective evaluation preoperative NLR, PLR and LMR are significantly associated with post-PNL SIRS. This is in concordance with the sparse literature [15, 18, 34] on the predictive value of these biomarkers in stone disease. The presence of stone causes release of inflammatory mediators suchas IL-6, IL-7, IL-8, TNF-α and GCSF, causing increased neutrophil counts. These accumulated cytokines in the tissue microenvironment provides an adequate environment for further stone formation [34]. Exaggerated inflammatory response suppresses the immune response by decreasing the cytolytic activity of lymphocytes, T cells and natural killer cells [34, 36]. Platelets are rich in proinflammatory agents and are capable of releasing active inflammatory metabolites [37]. Monocytes are also key regulators in systemic inflammatory response [38] Therefore, an increased preoperative NLR and PLR and decreased LMR can be indicative of an ongoing inflammatory reaction. These markers derived from complete blood count have become part of routine evaluation and are inexpensive. Higher NLR, PLR and lower LMR can be used as indicator for predicting SIRS and urosepsis, especially with negative urine cultures. Active anti-infective treatment during the perioperative period is vital to prevent SIRS and its progression to urosepsis.

Females have been known to have a higher incidence of infective complications post-PNL [39–41] as also seen in this study, although not being statistically significant. Low hemoglobin was significantly associated with postoperative SIRS which is in concordance with the available literature [39]. Diabetes mellitus has been independently related to post-PNL SIRS [41] as also seen in this study. Commonly reported risk factors for postoperative SIRS such as stone size, number of tracts, blood transfusion [7, 15, 16] which increase the complexity of the procedure and increased irrigation time were not found to be significantly associated in this patient cohort. Stag horn stones harbour colonized bacteria making preoperative urine sterilisation difficult therefore are commonly associated with postoperative sepsis [42] as seen in this study. Hard stones increase the operative time and both these factors were significantly associated with postoperative SIRS in this study as also reported in literature [43].

Other biomarkers such as C-reactive protein (CRP), erythrocyte sediment rate (ESR) procalcitonin and CRP/albumin ratio [39, 44, 45] have been reported as useful predictors of postoperative infection. But added costs do not allow them to be incorporated in the routine preoperative evaluation. NLR can be easily derived from peripheral blood cell count and not only forms a useful infective marker, but also related to sepsis severity [46]. Hemoglobin (anemia) is another such easily available cost-effective predictor of SIRS [18, 39], but the sensitivity and specificity of NLR, PLR and LMR in predicting postoperative SIRS have been proven to be much better than those of low hemoglobin. Different cutoff values have been suggested in multiple reports studying bacteremia, ICU stay or postoperative mortality [15, 34, 39, 47, 48]. This lack of consensus for a common value has limited the application of these hematologic markers in clinical practice and therefore requires more comprehensive prospective multi-institutional studies for improved evidence and standardization.

## Conclusion

Pathogenesis of postoperative infection is multifactorial and NLR, PLR and LMR can be useful independent, easily accessible and cost-effective predictors for post-PNL SIRS/sepsis. Patients with NLR > 2.03, PLR > 110.62 and LMR < 3.23 should be carefully followed for early identification of postoperative infective complications. More prospective studies are required to compare the accuracy of these biomarkers with the other risk factors to strengthen its clinical validation.
